# PERCEIVED STRESS AS A MEDIATOR OF THE ASSOCIATION BETWEEN PSYCHOLOGICAL RESILIENCE AND FUNCTIONAL DECLINES

**DOI:** 10.1093/geroni/igae098.2769

**Published:** 2024-12-31

**Authors:** Eunsaem Kim, Yunhwan Lee, Miji Kim, Sukyung Kim, YooGyung Shin, Jimin Han

**Affiliations:** Ajou University School of Medicine, Suwon-si, Kyonggi-do, Republic of Korea; Ajou University School of Medicine, Suwon, Kyonggi-do, Republic of Korea; Kyung Hee University, Seoul, Republic of Korea; Ajou University School of Medicine, Suwon, Kyonggi-do, Republic of Korea; Ajou University School of Medicine, Suwon, Kyonggi-do, Republic of Korea; Ajou University School of Medicine, Suwon, Kyonggi-do, Republic of Korea

## Abstract

Because older adults are highly likely to experience physical and functional losses during the aging process, resilience, the ability to cope with age-related stress and navigate adversities, is key to healthy aging. Although a growing number of studies have acknowledged the protective effects of psychological resilience on the onset of frailty, cognitive impairment, and physical disability, little is known about underlying mechanisms explaining the association between psychological resilience and functional declines in later life. To explore mediators that account for the path between psychological resilience and frailty, we used data from the 2023 Korean Frailty and Aging Cohort Study (KFACS), with 1397 aged 72-90 (Mean = 82.2). Mediating effects of perceived stress, cognitive function, and physical functioning were examined by a parallel-serial mediation model using PROCESS macro. Older adults with high psychological resilience were less likely to develop frailty (β = -.093, p <.05) after controlling for socio-demographic covariates and health-related factors. Psychological resilience influenced levels of frailty indirectly through perceived stress (β = -.04, 95% CI = -.078, -.002) and physical functioning (β = -.031, 95% CI = -.056, -.009). Also, there were simultaneous indirect effects of cognitive appraisal of stress through both cognitive function (β =-.01, 95% CI = -.019, -.003) and physical functioning (β = -.034, 95% CI = -.05, -.021) in the association between psychological resilience and frailty. These findings suggest that stress-coping abilities of older adults play a crucial role in promoting favorable stressor appraisals, which may mitigate functional declines.

